# Quantification of Y RNAs in peripheral blood mononuclear cells from patients with ankylosing spondylitis and healthy controls

**DOI:** 10.31744/einstein_journal/2026AO2319

**Published:** 2026-09-23

**Authors:** Guilherme Castellani, Matheus Braga, Aléia Harumi Uchibaba Yamanaka, Giovana Paola Zaccarias Bemvides, Julyane Schavaren, Larissa Danielle Bahls Pinto, Quirino Alves de Lima

**Affiliations:** 1 Post-Graduation Program in Biosciences and Physiopathology Department of Clinical Analysis and Biomedicine Universidade Estadual de Maringá Maringá PR Brazil Post-Graduation Program in Biosciences and Physiopathology, Department of Clinical Analysis and Biomedicine, Universidade Estadual de Maringá, Maringá, PR, Brazil.; 2 Immunogenetics Laboratory Department of Basic Health Sciences Universidade Estadual de Maringá Maringá PR Brazil Immunogenetics Laboratory, Department of Basic Health Sciences, Universidade Estadual de Maringá, Maringá, PR, Brazil.

**Keywords:** Autoimmune diseases, Inflammation, Blood cells, Quantitative real-time polymerase chain reaction, Biomarkers

## Abstract

**Objective:**

To investigate the expression of Y1, Y3, Y4, and Y5 in peripheral blood mononuclear cells from patients with ankylosing spondylitis and healthy controls.

**Methods:**

This case-control study included 10 patients with ankylosing spondylitis and 18 healthy controls from northern and northwestern Paraná, Brazil. The diagnosis of ankylosing spondylitis was confirmed by a rheumatologist according to the 2009 and 2011 ASAS criteria. Total RNA was extracted from peripheral blood using TRIzol reagent, treated with DNase I, and reverse-transcribed into cDNA. Y RNA expression was quantified by qPCR using SYBR™ Green, and the data were analyzed using Thermo Fisher Cloud.

**Results:**

Overall Y RNA expression did not differ significantly between patients and controls; however, lower ΔCt values, indicating higher Y1 and Y5 expression, were significantly associated with higher Bath Ankylosing Spondylitis Disease Activity Index (BASDAI) scores. Pairwise comparisons showed that Y3 and Y4 were expressed at lower levels than Y1 and that Y4 expression was lower than Y3 and Y5 expression; no significant differences were observed between Y1 and Y5 or between Y3 and Y5 in either patients or controls.

**Conclusion:**

These findings suggest that Y1 and Y5 expression is associated with disease activity, although no statistically significant differences in overall Y RNA expression were observed between patients and controls.

## Highlights

■ No significant difference in Y RNA expression was observed between patients with ankylosing spondylitis and controls.

■ Y1 and Y5 expression correlated with disease activity, as assessed using the BASDAI.

■ Y RNA expression was quantified by qPCR and analyzed using the 2−ΔΔCt method.

■ Y RNAs may be associated with inflammatory burden in ankylosing spondylitis.

## In Brief

Y RNA expression in peripheral blood mononuclear cells did not differ significantly between patients with ankylosing spondylitis and healthy controls. However, higher Y1 and Y5 expression was associated with greater disease activity, as assessed using the BASDAI, suggesting that these Y RNAs may warrant further investigation as candidate indicators of inflammatory burden.

## Graphical abstract

**Figure:**
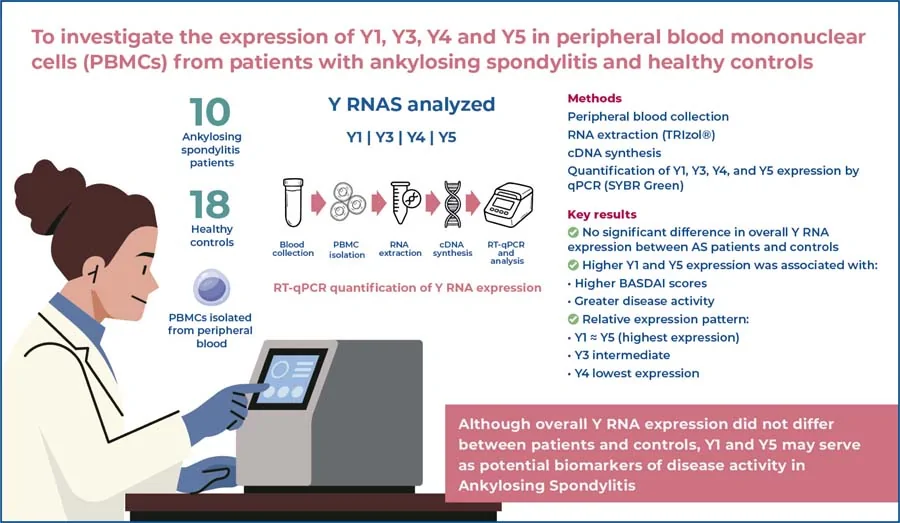

## INTRODUCTION

Spondyloarthritis (SpA) encompasses a group of diseases with shared clinical manifestations and is classified according to axial or peripheral symptoms. Axial forms involve inflammation of the sacroiliac joints and/or spine, whereas peripheral forms include arthritis, enthesitis, and dactylitis.^(1)^ Spondyloarthritis subtypes include ankylosing spondylitis (AS), psoriatic arthritis, arthritis associated with ulcerative colitis or Crohn’s disease, reactive arthritis, and undifferentiated spondyloarthritis.^(1,2)^ Ankylosing spondylitis is the most prevalent form of SpA and primarily affects Caucasian males from the third decade of life.^(1,3)^ Ankylosing spondylitis is characterized by joint inflammation, bone involvement, and impairment of the peripheral joints, particularly in the lower limbs, as well as the lumbar spine and sacroiliac joints. In severe cases, AS can lead to axial skeletal stiffness and functional limitations.^(1)^

The association between HLA-B*27 and AS is well established, with 90–95% of Caucasian patients with AS testing positive for this allele.^(4)^ However, in Brazil, the prevalence among patients with AS is significantly lower, suggesting genetic differences across populations.^(5)^ Nonetheless, HLA-B*27 alone is insufficient to determine AS development,^(6)^ and disease pathogenesis remains incompletely understood. Recent studies have highlighted the role of non-coding RNAs (ncRNAs) in various diseases, including AS.^7^ Among these ncRNAs, Y RNAs and their fragments have been described as bioactive molecules with biomarker potential, particularly in certain cancers.^10^

Y RNAs are transcribed by RNA polymerase III in the nucleus. In humans, four types have been identified: Y1, Y3, Y4, and Y5, with 113, 102, 96, and 84 nucleotides, respectively. These mature RNAs form characteristic hairpin structures through 3’ and 5’ pairing, with two main stems separated by a single-stranded pyrimidine-rich loop and distinct secondary structures.^(8)^ At the 3’ end, an oligo-uridylated sequence enables interaction with the La protein, protecting against 3’–5’ exonuclease degradation and promoting nuclear retention.^(8)^ Y RNAs also associate with Ro ribonucleoproteins, forming Ro-RNP complexes.^(13,14)^ However, whether Y RNAs remain bound to La during export or bind after translocation remains unclear.^(8)^ Functionally, Y RNAs are involved in DNA replication, regulation of mRNA stability, and cellular stress responses.^(8,15,16)^

Given their reported roles in immune responses and disease mechanisms, Y RNAs may be implicated in AS pathogenesis. However, few studies have explored this connection. Investigating the expression of Y1, Y3, Y4, and Y5 RNAs in patients with AS may help clarify whether these transcripts are associated with disease activity and may support their further evaluation as candidate molecular biomarkers. Because Y RNAs have been proposed as biomarkers in other conditions, their quantification in AS may provide clinically relevant preliminary information. Such findings may contribute to a better understanding of molecular features associated with AS and inform future studies on diagnostic or monitoring approaches.

## OBJECTIVE

This study aimed to quantify Y RNA expression in patients with ankylosing spondylitis and healthy controls using qPCR.

## METHODS

### Study design and participants

This case-control study evaluated patients diagnosed with AS (n=10) and individuals without AS (control group; n=18), all from the northern and northwestern regions of the state of Paraná. Patients with AS were recruited from the Rocha Loures Rheumatology Clinic, which serves patients from the Maringá region. A qualified rheumatologist diagnosed AS based on the 2009 Assessment of SpondyloArthritis International Society (ASAS) criteria for axial spondyloarthritis^(17)^ and the 2011 ASAS criteria for peripheral spondyloarthritis.^(18)^

The Control Group comprised individuals with no history of rheumatologic or autoimmune diseases who were selected from employees undergoing routine health assessments at the Teaching, Research, and Clinical Analysis Laboratory (LEPAC) of the *Universidade Estadual de Maringá*. All participants were older than 18 years, and both the patient and Control Groups consisted of unrelated individuals.

### Ethics approval and consent to participate

This study was approved by the Permanent Ethics Committee of the *Universidade Estadual de Maringá* (CAAE: 27723114.0.0000.0104; # 5.231.082). Written informed consent was obtained from all participants included in the study.

### Blood sample collection and peripheral blood mononuclear cell isolation

Peripheral blood was collected in EDTA-containing anticoagulant tubes and processed within 2 hours to prevent RNA degradation. peripheral blood mononuclear cells (PBMCs) were isolated by density-gradient centrifugation using Ficoll-Hypaque (Sigma-Aldrich) according to the manufacturer’s instructions.

### Total RNA extraction and quantification

Total RNA was extracted from the isolated cells using TRIzol^®^ LS reagent (Invitrogen Life Technologies), with an adapted protocol that included precipitation with isopropanol and washing with 80% ethanol. The extracted RNA samples were resuspended in RNase-free water with 10% ammonium sulfate (10M) and stored at -80°C for future testing. Total RNA concentration was assessed by measuring optical density at 260 and 280nm using a NanoDrop 2000^®^ spectrophotometer (Thermo Scientific).

To evaluate RNA quality, an aliquot was subjected to electrophoresis on a 1% agarose gel stained with ethidium bromide. After electrophoresis, a characteristic RNA smear was observed, along with two distinct bands corresponding to 18S and 28S ribosomal RNA. Following extraction, the RNA samples were treated with a DNase I/RNase-free kit (Thermo Scientific) to digest single- or double-stranded DNA. This step was performed before quantitative reverse transcription polymerase chain reaction (RT-qPCR) because DNA is considered a contaminant in this context.

### cDNA library construction

The purified total RNA was used as a template for cDNA synthesis. Random primers and reverse transcriptase enzyme (Thermo Scientific) were used for this process. Following cDNA synthesis, PCR using GAPDH primers was performed to confirm cDNA viability before subsequent analysis. The products were separated by electrophoresis on a 2% agarose gel stained with SYBR™ Safe DNA Gel Stain (Invitrogen Life Technologies).

### cDNA quantification with PowerUp™ SYBR™ Green Master Mix

cDNA quantification was performed by quantitative polymerase chain reaction (qPCR) using the PowerUp™ SYBR™ Green Master Mix kit (Thermo Scientific) for *GAPDH*, used as the reference gene, and all *RNY* genes. Initial sample quantity was determined by monitoring fluorescence emission at each cycle. Information on all primer pairs is provided in Table 1S, Supplementary Material. Nonspecific amplification and potential secondary structures were checked using the Basic Local Alignment Search Tool (http://www.ncbi.nlm.nih.gov/tools/primer-blast) and Oligo Analyzer 7 software (Integrated DNA Technologies, United States).

qPCR amplification was performed in triplicate in a total volume of 10μL containing 5ng of cDNA, 600nM of each primer, and 1× PowerUp™ SYBR™ Green Master Mix (Applied Biosystems™). The reactions were run on the StepOnePlus™ System (Thermo Scientific) under the following conditions: 2 min at 50°C; 2 min at 95°C; and 40 cycles of 15s at 95°C, 15s at 58°C, and 30s at 72°C. Melting curve analysis was performed under the following conditions: 25s at 95°C, 1 min at 60°C, and 15s at 95°C.

### Statistical analysis

Cq, ΔCt, ΔΔCt, and relative quantification (RQ) values were obtained using the Thermo Fisher Cloud platform. Subsequent statistical analyses were performed in R (version 4.5.1; R Core Team). The comparative cycle threshold (Ct) method was used to present relative gene expression across samples, and fold change was calculated using the 2^Ct^ method.^(19)^

Data distribution was evaluated using the Shapiro–Wilk test. Depending on normality, comparisons between patients and controls were performed using Student’s *t*-test or the Mann–Whitney *U* test. For multiple within-group comparisons of ΔCt values across genes, the Friedman test was applied, followed by post hoc pairwise Wilcoxon signed-rank tests with Bonferroni adjustment.

Linear regression analyses were conducted to investigate associations between ΔCt values and disease activity, as measured by the Bath Ankylosing Spondylitis Disease Activity Index (BASDAI). The coefficient of determination (R^2^) and p values were reported for each model. Because gene expression values were expressed as ΔCt, lower ΔCt values corresponded to higher relative gene expression levels, a p<0.05 was considered statistically significant.

## RESULTS

No significant differences in age or sex distribution were observed between controls and patients with AS (Table 1).

**Table 1:** Demographic and clinical characteristics of study participants

	Control Group n=18	AS Group n=10	p value
Age (years)	42.23±18.05	47.00±9.91	0.38^*^
BASDAI	-	5.44±2.34	-
Sex, n (%)			
Female	8 (44)	6 (60)	0.69^#^
Male	10 (56)	4 (40)	

Data are presented as mean±standard deviation (SD) unless otherwise indicated. *Student’s t-test; #chi-squared (χ^2^) test.

AS: ankylosing spondylitis; BASDAI: Bath Ankylosing Spondylitis Disease Activity Index.

Analysis of ΔCt values showed no statistically significant differences between patients and controls for the evaluated genes (Table 2). All individual Cq and ΔCt values for each gene are provided in Table 2S, Supplementary Material. Relative expression, expressed as RQ, was calculated using the 2^Ct^ method, normalized to a value of 1.00 in controls, and is shown in figure 1. Patients showed numerically higher expression of Y1, Y3, and Y5 and numerically lower expression of Y4. However, these differences did not reach statistical significance.

**Table 2:** Relative quantification values in controls and patients with ankylosing spondylitis (median, min–max)

Gene	RQ (min–max), controls	RQ (min–max), AS	p value^*^
Y1	1 (0.68-1.45)	2.23 (0.73-6.80)	0.27^#^
Y3	1 (0.55-1.81)	1.47 (1.38-5.69)	0.21^$^
Y4	1 (0.35-2.78)	0.59 (0.14-2.44)	0.92^#^
Y5	1 (0.59-1.67)	2.24 (0.65-7.71)	0.20^$^

*p values were calculated using threshold cycle differences (ΔCt values); #Mann–Whitney U test; $Student’s t-test.

AS: ankylosing spondylitis; ΔCt: delta threshold cycle; RQ: relative quantification.

Controls were normalised to 1.00. Error bars represent. No statistically significant between-group differences were observed for any transcript.

**Figure 1:**
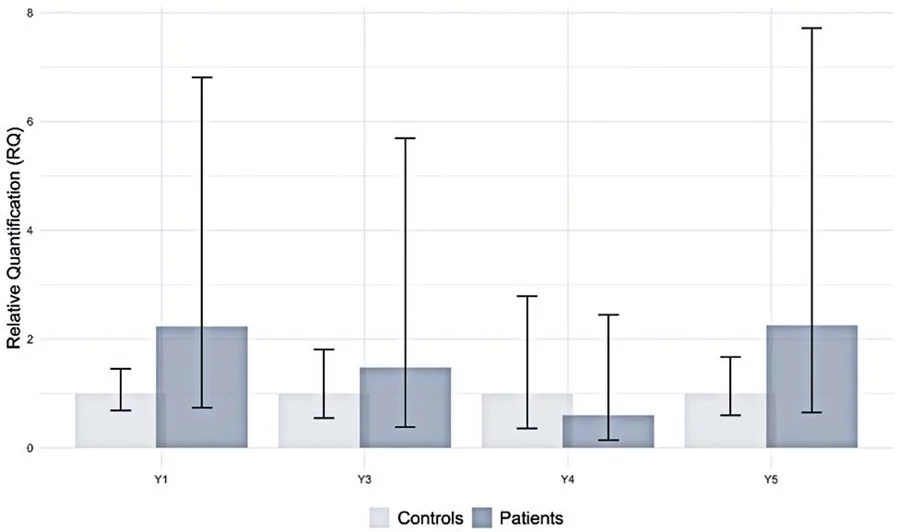
Relative gene expression of Y1, Y3, Y4, and Y5 in controls and patients with ankylosing spondylitis, analysed using the 2−ΔΔCt method

Linear regression analysis in patients showed that lower ΔCt values, indicating higher gene expression, were significantly associated with higher BASDAI scores for Y1 (p=0.04) and Y5 (p=0.01). No significant association was observed for Y3 (p=0.84) or Y4 (p=0.96). These results indicate that higher disease activity was associated with higher Y1 and Y5 expression. These relationships are shown in figure 2, where the inverse association between ΔCt values and BASDAI scores is illustrated.

**Figure 2:**
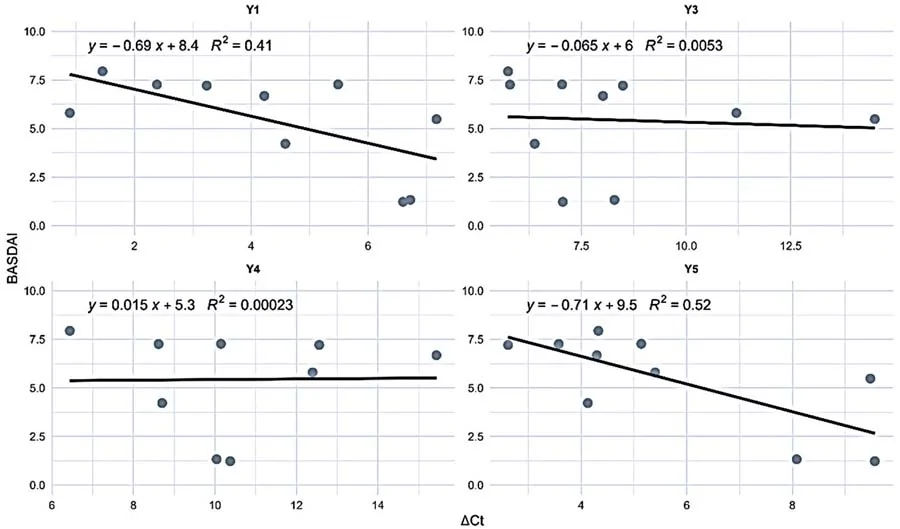
Association between Y RNA expression and disease activity in patients with ankylosing spondylitis

Scatter plots show ΔCt values for Y1, Y3, Y4, and Y5 plotted against BASDAI scores, with linear regression lines and R^2^ values. Lower ΔCt values indicate higher gene expression. Significant associations were observed for Y1 and Y5, whereas no significant associations were observed for Y3 or Y4.

Analysis of ΔCt values revealed significant differences in gene expression among patients (Friedman test, χ^2^=23.13, df=3, p <0.001). Post hoc comparisons showed that Y3 and Y4 were expressed at significantly lower levels than Y1 and that Y4 was expressed at lower levels than Y3 and Y5; no significant differences were observed between Y1 and Y5 or between Y3 and Y5 (Table 3, Figure 3).

**Table 3:** Post hoc pairwise comparisons of Y RNA expression (ΔCt-based) in patients with ankylosing spondylitis, Wilcoxon signed-rank test with Bonferroni correction

Comparison	Mean RQ (normalised to Y1=1)	p value*	Bonferroni-adjusted p value
Y1–Y3	1-0.06	<0.01	**0.02**
Y1–Y4	1-0.01	<0.01	0.02
Y1–Y5	1-0.38	0.12	0.77
Y3–Y4	0.06-0.01	<0.01	0.02
Y3–Y5	0.06-0.38	0.05	0.32
Y4–Y5	0.01-0.38	<0.01	0.02

*Raw p values calculated using threshold cycle differences (ΔCt values), Wilcoxon signed-rank test. Bold Bonferroni-corrected p values indicate p<0.05.

ΔCt: delta threshold cycle; RQ: relative quantification.

**Figure 3:**
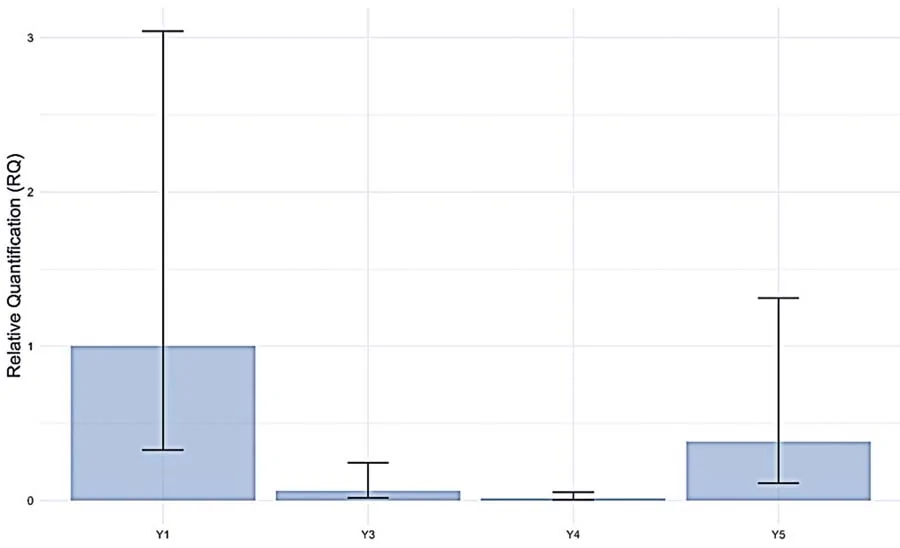
Relative Y RNA expression in patients with ankylosing spondylitis, analysed using the 2−ΔΔCt method

Relative quantification values are shown for patients with AS (n=10). Error bars represent RQ min – RQ max. Pairwise comparisons were performed on ΔCt values with Bonferroni correction.

Analysis of ΔCt values in controls revealed significant differences in gene expression (Friedman test, χ^2^=38.01, df=3, p<0.001). Post hoc comparisons showed that Y3 and Y4 were expressed at significantly lower levels than Y1 and that Y4 was expressed at lower levels than Y3 and Y5; no significant differences were observed between Y1 and Y5 or between Y3 and Y5 (Table 4, Figure 4).

**Table 4:** Post hoc pairwise comparisons of Y RNA expression (ΔCt-based) in healthy controls, Wilcoxon signed-rank test with Bonferroni correction

Comparison	Median RQ (normalised to Y1=1)	p value*	Bonferroni-adjusted p value
Y1–Y3	1-0.09	<0.01	<0.01
Y1–Y4	1-0.04	<0.01	<0.01
Y1–Y5	1-0.38	<0.01	<0.01
Y3–Y4	0.09-0.04	0.07	0.40
Y3–Y5	0.09-0.38	<0.01	<0.01
Y4–Y5	0.04-0.38	<0.01	0.01

*Raw p values calculated using threshold cycle differences (ΔCt values), Wilcoxon signed-rank test. Bold Bonferroni-corrected p values indicate p<0.05.

ΔCt: delta threshold cycle; RQ: relative quantification.

**Figure 4:**
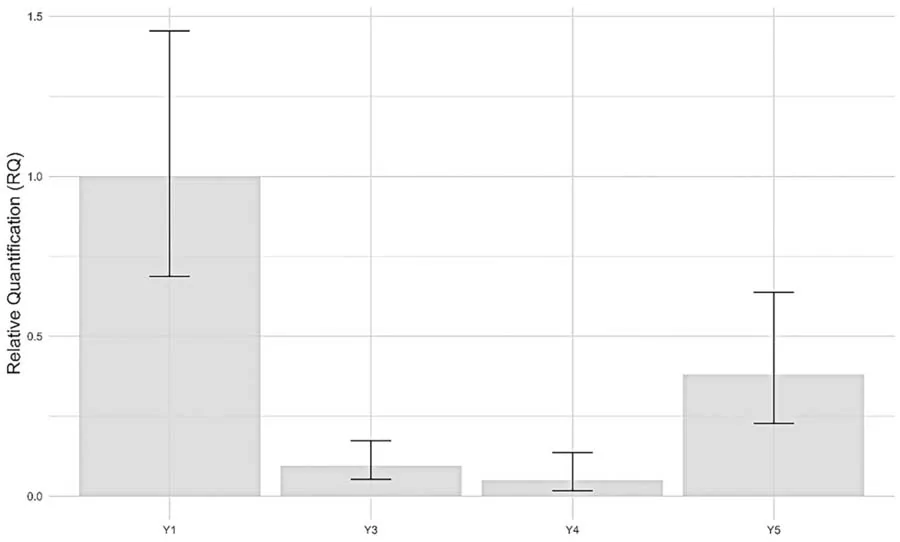
Relative Y RNA expression in healthy controls, analysed using the 2−ΔΔCt method

Relative quantification (RQ) values are shown for healthy controls (n=18). Error bars represent RQ min – RQ max. Pairwise comparisons were performed on ΔCt values with Bonferroni correction.

## DISCUSSION

This study examined Y RNA expression in PBMCs from patients with AS and healthy controls. No statistically significant between-group differences in overall expression were observed. Two main findings emerged: Y1 and Y5 were the most abundantly expressed Y RNA transcripts, and higher expression of both was significantly associated with higher disease activity as measured by the BASDAI. These observations suggest that specific Y RNA transcripts may be associated with disease activity in AS.

To our knowledge, this is among the first studies to investigate Y RNA expression in AS. Previous investigations have mainly focused on the role of Y RNAs in other autoimmune conditions, particularly systemic lupus erythematosus and Sjögren’s syndrome. In these diseases, Y RNAs are recognised as structural components of Ro60/La ribonucleoprotein complexes that participate in RNA quality control and cellular stress responses. These complexes are thought to contribute to immune regulation by stabilising non-coding RNAs and protecting misfolded RNAs from degradation, thereby helping to maintain RNA homeostasis under cellular stress.^(20,21)^

The association between Y1 and Y5 expression and disease activity is another noteworthy finding. Linear regression analyses showed that lower ΔCt values — corresponding to higher gene expression — were significantly associated with higher BASDAI scores. These findings raise the possibility that Y RNA expression may be related to inflammatory activity in AS. Fragments derived from Y RNAs, known as Y RNA-derived small RNAs (s-RNYs), have been described as biologically active molecules that can modulate immune responses. Experimental evidence indicates that s-RNYs may promote inflammatory and apoptotic pathways in monocytes and macrophages, which may be functionally linked to immune activation in AS.^(22)^

A differential expression pattern was also observed among Y RNA family members. In both patients and controls, Y1 and Y5 were the most abundantly expressed transcripts, whereas Y3 and, particularly, Y4 showed lower expression levels. Y RNA family members are known to differ in transcriptional regulation, intracellular stability, and association with RNA-binding proteins, which may account for differences in abundance and functional roles.^(23,24)^Y1 and Y5 are frequently enriched in ribonucleoprotein complexes and have been implicated in RNA surveillance pathways and cellular stress responses; Y4, by contrast, has been reported to be a less abundant transcript in several cell types, possibly reflecting lower transcriptional activity or reduced RNA stability.^23,24)^

The biological mechanisms linking Y RNAs to AS remain incompletely understood. However, several pathways relevant to disease pathogenesis may intersect with Y RNA biology. RNA turnover and stability are regulated by RNA-binding proteins and non-coding RNAs, and disturbances in these processes may influence inflammatory responses.^(25)^Non-coding RNAs have increasingly been implicated in the regulation of innate and adaptive immune pathways in autoimmune diseases, including cytokine signalling, leucocyte activation, and the maintenance of inflammatory states.^(26)^ Recent advances in RNA immunobiology further support the concept that RNA species and their processing products can shape immune responses rather than merely representing by-products of cellular metabolism.^(27)^

The observed association between Y RNA expression and disease activity raises the possibility that Y1 and Y5 may warrant further investigation as candidate indicators of inflammatory burden in AS. Non-coding RNAs have been investigated as potential biomarkers because of their relative stability and detectability in peripheral blood cells, extracellular vesicles, and other biological fluids.^(28)^ Similar approaches have been proposed for autoimmune diseases, in which circulating and cell-associated non-coding RNAs may provide information relevant to disease status and immune dysregulation.^(29)^Small circulating non-coding RNAs have also been explored as biomarkers in other pathological settings, further supporting their potential translational relevance.^(30)^Although this study did not identify significant between-group differences in overall expression, the association between Y1 and Y5 expression and BASDAI scores suggests that these transcripts may provide preliminary information regarding disease activity in AS.

Several limitations should be acknowledged. First, the small sample size may have limited statistical power to detect subtle between-group differences in Y RNA expression, increasing the risk of type II error. The regression analyses were also based on a limited number of patients, making the observed associations susceptible to individual variability and outlier effects. Second, gene expression was evaluated exclusively in PBMCs, which may not fully reflect molecular processes occurring in disease-relevant tissues such as the sacroiliac joints or spinal entheses. Third, the cross-sectional design precludes evaluation of temporal changes in Y RNA expression during disease progression or in response to treatment. Longitudinal studies are needed to determine whether Y RNA expression or derived fragment levels change over time and whether they may be associated with therapeutic outcomes.

Future studies with larger patient cohorts are needed to validate the associations reported here. Investigations of Y RNA expression in disease-relevant tissues, together with mechanistic studies examining interactions between Y RNAs and inflammatory signaling pathways, may provide further insight into their potential role in spondyloarthritis.

## CONCLUSION

Among the Y RNAs analyzed, Y1 was the most highly expressed transcript in both patients with ankylosing spondylitis and healthy controls. Although no significant between-group differences were observed, higher Y1 and Y5 expression was associated with greater disease activity as measured by the BASDAI. These findings raise the possibility that Y RNA expression is associated with disease activity in ankylosing spondylitis. Further studies with larger cohorts, longitudinal follow-up, and tissue-specific analyses are warranted to clarify the functional relevance of Y RNAs and their derived fragments in spondyloarthritis.

## SUPPLEMENTARY MATERIAL

Supplementary Material
