# Supplementary material for: Quantification of Y RNAs in peripheral blood mononuclear cells from patients with ankylosing spondylitis and healthy controls
Source: Einstein (Sao Paulo). 2026 Sep 23;24:eAO2319. doi: 10.31744/einstein_journal/2026AO2319 (PMC13682767; doi:10.31744/einstein_journal/2026AO2319)
Supplement: Supplementary file 1 [file 2317-6385-eins-24-eAO2319-suppl01.pdf]

SUPPLEMENTARY MATERIAL

Quantification of Y RNAs in peripheral blood mononuclear cells from patients with ankylosing spondylitis and healthy controls

Guilherme Castellani, Matheus Braga, Aléia Harumi Uchibaba Yamanaka, Giovana Paola Zaccarias Bemvides, Julyane Schavaren, Larissa Danielle Bahls Pinto, Quirino Alves de Lima Neto

DOI: 10.31744/einstein_journal/2026AO2319

Table 1S. Oligonucleotide primers used for RT-qPCR quantification of *GAPDH*, *RNY1*, *RNY3*, *RNY4*, and *RNY5*

Gene		Primer Sequences 5′ to 3′
GAPDH	F	AGGTGAAGGTCGGAGTCAAC
	R	CCATGTAGTTGAGGTCAATGAAG
RNY1	F	GGC TGG TCC GAA GGT AGT GA
	R	AAA AGA CTA GTC AAG TGG AG
RNY3	F	GGC TGG TCC GAG TGC AGT GG
	R	AAA AGG CTA GTC AAG CAG TGG G
RNY4	F	GGC TGG TCC GAT GGT AGT GG
	R	AAA AAG CCA GTC AAA TTT AG
RNY5	F	AGT TGG TCC GAG TGT TGT GG
	R	AAA ACA GCA AGC TAG TCA AG

F: forward primer; R: reverse primer. Primer sequences are shown in the 5′→3′ direction.

Table 2S. Individual RT-qPCR Cq and ΔCt values for *GAPDH*, *RNY1*, *RNY3*, *RNY4*, and *RNY5* in patients with ankylosing spondylitis and healthy controls

Sample	Group	Target	Mean Cq	ΔCt	ΔCt.SE	F.Factor
CG01	Control	*GAPDH*	23.287	-	-	-
CG01	Control	Y1	28.582	5.295	0.182	2.776
CG01	Control	Y3	32.528	9.241	0.502	2.447
CG01	Control	Y4	33.584	10.297	0.568	2.776
CG01	Control	Y5	29.897	6.61	0.369	2.776
CG02	Control	*GAPDH*	28.531	-	-	-
CG02	Control	Y1	32.845	4.315	0.333	2.776
CG02	Control	Y3	35.345	6.814	0.604	2.365
CG02	Control	Y4	29.375	0.844	0.519	2.776
CG02	Control	Y5	34.015	5.485	0.868	2.776
CG03	Control	*GAPDH*	24.444	-	-	-
CG03	Control	Y1	29.346	4.902	0.216	2.776
CG03	Control	Y3	33.99	9.547	0.376	2.447
CG03	Control	Y4	30.797	6.354	0.275	2.776
CG03	Control	Y5	31.375	6.931	0.393	2.776
CG04	Control	*GAPDH*	23.213	-	-	-
CG04	Control	Y1	29.691	6.478	0.151	2.776
CG04	Control	Y3	36.026	12.813	1.133	2.776
CG04	Control	Y4	36.048	12.836	0.228	2.776
CG04	Control	Y5	32.365	9.152	0.119	2.776

continue...

...Continuation

Table 2S. Individual RT-qPCR Cq and ΔCt values for *GAPDH*, *RNY1*, *RNY3*, *RNY4*, and *RNY5* in patients with ankylosing spondylitis and healthy controls

Sample	Group	Target	Mean Cq	ΔCt	ΔCt.SE	F.Factor
CG05	Control	*GAPDH*	21.087	-	-	-
CG05	Control	Y1	26.64	5.553	1.683	2.776
CG05	Control	Y3	28.398	7.311	0.174	2.776
CG05	Control	Y4	30.126	9.039	0.088	2.776
CG05	Control	Y5	25.963	4.876	0.232	2.776
CG06	Control	*GAPDH*	22.582	-	-	-
CG06	Control	Y1	28.98	6.398	0.461	2.776
CG06	Control	Y3	32.988	10.406	0.18	2.776
CG06	Control	Y4	34.021	11.438	0.383	2.776
CG06	Control	Y5	30.434	7.852	0.263	2.776
CG12	Control	*GAPDH*	27.836	-	-	-
CG12	Control	Y1	34.272	6.436	0.455	3.182
CG12	Control	Y3	35.427	7.592	0.425	2.776
CG12	Control	Y4	38.364	10.529	-	-
CG12	Control	Y5	33.858	6.023	0.34	2.776
CG14	Control	*GAPDH*	20.701	-	-	-
CG14	Control	Y1	25.966	5.265	0.279	4.303
CG14	Control	Y3	28.178	7.477	0.24	3.182
CG14	Control	Y4	31.106	10.405	0.616	3.182
CG14	Control	Y5	28.166	7.466	0.256	3.182
CG15	Control	*GAPDH*	23.062	-	-	-
CG15	Control	Y1	29.121	6.059	0.338	3.182
CG15	Control	Y3	33.236	10.174	0.466	2.776
CG15	Control	Y4	33.147	10.085	0.531	2.776
CG15	Control	Y5	29.947	6.885	0.368	3.182
CG16	Control	*GAPDH*	23.003	-	-	-
CG16	Control	Y1	28.036	5.033	0.228	2.776
CG16	Control	Y3	31.366	8.363	0.315	2.776
CG16	Control	Y4	-	-	-	-
CG16	Control	Y5	29.003	6.0	0.227	2.776
CG17	Control	*GAPDH*	22.547	-	-	-
CG17	Control	Y1	28.027	5.48	0.243	2.776
CG17	Control	Y3	32.382	9.835	0.257	2.776
CG17	Control	Y4	31.558	9.01	0.298	2.776
CG17	Control	Y5	29.2	6.653	0.401	2.776
CG18	Control	*GAPDH*	25.533	-	-	-
CG18	Control	Y1	27.572	2.039	0.342	2.776
CG18	Control	Y3	30.759	5.225	0.173	2.776
CG18	Control	Y4	34.982	9.449	0.761	2.776
CG18	Control	Y5	29.299	3.766	0.489	2.776
CG19	Control	*GAPDH*	21.544	-	-	-
CG19	Control	Y1	28.212	6.668	0.244	2.776
CG19	Control	Y3	32.267	10.723	0.146	2.776
CG19	Control	Y4	33.671	12.127	0.103	2.776
CG19	Control	Y5	31.098	9.554	0.447	2.776
CG20	Control	*GAPDH*	23.056	-	-	-
CG20	Control	Y1	28.635	5.579	0.277	2.776
CG20	Control	Y3	31.417	8.361	0.082	3.182

continue...

...Continuation

Table 2S. Individual RT-qPCR Cq and ΔCt values for *GAPDH*, *RNY1*, *RNY3*, *RNY4*, and *RNY5* in patients with ankylosing spondylitis and healthy controls

Sample	Group	Target	Mean Cq	ΔCt	ΔCt.SE	F.Factor
CG20	Control	Y4	32.153	9.097	0.14	2.776
CG20	Control	Y5	30.191	7.135	0.336	2.776
CG21	Control	*GAPDH*	22.499	-	-	-
CG21	Control	Y1	28.266	5.767	0.141	3.182
CG21	Control	Y3	30.894	8.395	0.255	2.776
CG21	Control	Y4	34.773	12.274	0.158	2.776
CG21	Control	Y5	28.683	6.184	0.082	2.776
CG22	Control	*GAPDH*	22.572	-	-	-
CG22	Control	Y1	29.182	6.61	0.362	2.776
CG22	Control	Y3	32.362	9.79	1.06	2.776
CG22	Control	Y4	35.22	12.647	0.855	3.182
CG22	Control	Y5	31.569	8.997	0.422	2.776
CG23	Control	*GAPDH*	22.587	-	-	-
CG23	Control	Y1	27.459	4.872	0.161	3.182
CG23	Control	Y3	31.26	8.673	0.252	4.303
CG23	Control	Y4	34.189	11.602	0.392	3.182
CG23	Control	Y5	30.279	7.692	0.44	3.182
CG24	Control	*GAPDH*	22.965	-	-	-
CG24	Control	Y1	28.068	5.102	-	-
CG24	Control	Y3	30.872	7.907	-	-
CG24	Control	Y4	31.266	8.301	-	-
CG24	Control	Y5	28.631	5.666	-	-
EA03	Patient	*GAPDH*	21.391	-	-	-
EA03	Patient	Y1	25.614	4.223	0.215	2.447
EA03	Patient	Y3	29.407	8.016	0.193	2.447
EA03	Patient	Y4	36.827	15.436	1.348	2.571
EA03	Patient	Y5	25.688	4.297	0.291	2.447
EA04	Patient	*GAPDH*	21.214	-	-	-
EA04	Patient	Y1	26.701	5.486	0.208	2.776
EA04	Patient	Y3	28.252	7.037	0.405	2.776
EA04	Patient	Y4	31.361	10.147	0.245	2.776
EA04	Patient	Y5	26.354	5.139	1.245	2.776
EA05	Patient	*GAPDH*	23.147	-	-	-
EA05	Patient	Y1	24.041	0.894	0.372	2.776
EA05	Patient	Y3	34.346	11.2	0.368	2.776
EA05	Patient	Y4	35.543	12.396	0.752	2.776
EA05	Patient	Y5	28.546	5.4	3.009	2.776
EA06	Patient	*GAPDH*	26.069	-	-	-
EA06	Patient	Y1	28.455	2.386	0.157	2.776
EA06	Patient	Y3	31.86	5.792	0.384	2.776
EA06	Patient	Y4	34.686	8.618	0.314	2.776
EA06	Patient	Y5	29.645	3.576	0.688	2.776
EA07	Patient	*GAPDH*	22.979	-	-	-
EA07	Patient	Y1	29.7	6.721	0.229	2.776
EA07	Patient	Y3	31.269	8.289	0.138	3.182
EA07	Patient	Y4	33.021	10.041	0.101	2.776
EA07	Patient	Y5	31.062	8.082	0.312	2.776
EA08	Patient	*GAPDH*	22.538	-	-	-
EA08	Patient	Y1	25.775	3.237	0.194	2.776

continue...

...Continuation

Table 2S. Individual RT-qPCR Cq and ΔCt values for *GAPDH*, *RNY1*, *RNY3*, *RNY4*, and *RNY5* in patients with ankylosing spondylitis and healthy controls

Sample	Group	Target	Mean Cq	ΔCt	ΔCt.SE	F.Factor
EA08	Patient	Y3	31.028	8.49	2.828	2.776
EA08	Patient	Y4	35.097	12.559	0.677	2.776
EA08	Patient	Y5	25.155	2.617	0.183	2.776
EA09	Patient	*GAPDH*	21.512	-	-	-
EA09	Patient	Y1	28.68	7.169	0.205	2.776
EA09	Patient	Y3	36.019	14.507	1.987	3.182
EA09	Patient	Y4	-	-	-	-
EA09	Patient	Y5	30.987	9.476	0.375	2.776
EA10	Patient	*GAPDH*	22.191	-	-	-
EA10	Patient	Y1	28.788	6.597	0.932	2.776
EA10	Patient	Y3	29.248	7.057	0.469	3.182
EA10	Patient	Y4	32.566	10.375	0.852	2.776
EA10	Patient	Y5	31.751	9.56	0.708	2.776
EA11	Patient	*GAPDH*	24.703	-	-	-
EA11	Patient	Y1	26.156	1.453	0.264	3.182
EA11	Patient	Y3	30.452	5.748	0.289	2.776
EA11	Patient	Y4	31.142	6.439	0.566	2.776
EA11	Patient	Y5	29.029	4.326	0.093	2.776
EA12	Patient	*GAPDH*	23.897	-	-	-
EA12	Patient	Y1	28.479	4.582	1.006	3.182
EA12	Patient	Y3	30.28	6.383	0.924	3.182
EA12	Patient	Y4	32.601	8.704	0.914	3.182
EA12	Patient	Y5	28.023	4.126	0.9	2.776

Sample: sample identifier; group: experimental group (control or ankylosing spondylitis); Target: gene analyzed; Mean Cq: mean quantification cycle; ΔCt: difference between the Cq of the target gene and the reference gene; ΔCt.SE: standard error of ΔCt; F.Factor: efficiency correction factor.
